# Radiologically Occult Desmoplastic Small Round Cell Tumor in a 36-Year-Old Man: A Case Report

**DOI:** 10.7759/cureus.101223

**Published:** 2026-01-10

**Authors:** Yassamin Benhayoun Sadafyine, Fatima Belabbes, Nawal Bouknani, Nabil Benjelloun, Imane Ben elbarhdadi

**Affiliations:** 1 Gastroenterology, Mohammed VI University of Health and Sciences, Casablanca, MAR; 2 Gastroenterology and Proctology, Cheikh Khalifa International University Hospital, Casablanca, MAR; 3 Gastroenterology and Proctology, Mohammed VI University of Health Sciences (UM6SS), Casablanca, MAR; 4 Radiology, Cheikh Khalifa International University Hospital, Casablanca, MAR; 5 Faculty of Medicine, Mohammed VI University of Health Sciences (UM6SS), Casablanca, MAR; 6 Gastroenterology, Cheikh Khalifa International University Hospital, Casablanca, MAR; 7 Gastroenterology and Hepatology, Faculty of Medicine, Mohammed VI University of Health Sciences (UM6SS), Casablanca, MAR

**Keywords:** abdominopelvic tumor, chemotherapy, desmoplastic small round cell tumor, liver metastasis, oncology, young male

## Abstract

Desmoplastic small round cell tumor (DSRCT) is a rare and aggressive mesenchymal malignancy that typically affects adolescents and young adults. We report an unusual case of a 36-year-old man who presented with persistent left flank pain. Initial investigations, including colonoscopy and abdominal computed tomography, were unremarkable. One year later, repeat imaging revealed a large intra-abdominal mass associated with liver metastases. Histopathological examination and immunohistochemical analysis confirmed the diagnosis of DSRCT. This case highlights the diagnostic challenges of DSRCT, particularly when it occurs outside the usual age range and presents with non-specific symptoms. Persistent or unexplained symptoms should prompt repeat and advanced imaging, as well as early tissue diagnosis, to facilitate timely multidisciplinary management.

## Introduction

Desmoplastic small round cell tumor (DSRCT) is a rare and highly aggressive mesenchymal malignancy characterized by the t(11;22)(p13;q12) chromosomal translocation, resulting in the Ewing Sarcoma Breakpoint Region 1, Wilms Tumor 1 (EWSR1-WT1) fusion gene [1]. First described by Gerald and Rosai in 1989 [2], DSRCT predominantly affects adolescent and young adult males, arising from peritoneal serosal surfaces. Clinical presentation often includes nonspecific abdominal pain, distension, palpable masses, or ascites. At diagnosis, the disease is frequently advanced, and outcomes remain poor despite aggressive multimodal therapy [3,4]. Diagnosis relies on histopathological examination revealing nests of small round blue cells within a prominent desmoplastic stroma, along with a characteristic polyphenotypic immunoprofile (epithelial markers such as cytokeratins/EMA, muscle differentiation with desmin in a perinuclear dot pattern, and nuclear WT1 positivity) and cytogenetic/molecular confirmation of the EWSR1-WT1 fusion [1,5]. We present an uncommon case of a 36-year-old man, 10 years beyond the average age (mean age: 20-22), with initially negative cross-sectional imaging and subsequent documentation of extensive intra-abdominal disease one year later.

This case underscores the potential for early false-negative imaging in DSRCT, the need for repeated evaluation when symptoms persist, and the central role of tissue diagnosis with ancillary molecular testing to enable timely management.

## Case presentation

A 36-year-old man with no significant past medical history presented to our Gastroenterology Department with intermittent left flank pain persisting for several weeks. His general condition was preserved. No anorexia, no fever, no nausea, or other functional signs was reported. 

On physical examination, his abdominal perimeter was within normal limits. There was no ascites, abdominal wall defense, palpable mass, or organomegaly. No peripheral lymphadenopathy was detected. The testicular examination was unremarkable, ruling out a potential differential diagnosis of a germ cell tumor. Palpation of the left flank elicited mild, localized tenderness without rebound or guarding. Laboratory tests were within normal limits (Table 1). Tumor markers were not assessed at this stage.

**Table 1:** Summary of laboratory test. CRP: C-reactive protein; LDH: lactate dehydrogenase; ALP: alkaline phosphatase; GGT: gamma-glutamyl transferase; BUN: blood urea nitrogen.

Test	Result	Reference range	Unit
Hemoglobin	14.2	13–17	g/dL
White blood cells	6,800	4,000–10,000	/µL
Platelets	245,000	150,000–400,000	/µL
CRP	3	<5	mg/L
LDH	180	120–250	U/L
ALP	78	40–130	U/L
GGT	22	10–50	U/L
Total bilirubin	0.8	0.2–1.2	mg/dL
Albumin	42	35–50	g/L
Serum creatinine	0.9	0.6–1.2	mg/dL
BUN	13	7–20	mg/dL

A colonoscopy was performed to rule out colonic pathology. The examination was unremarkable, with a normal appearing colonic mucosa throughout. Contrast-enhanced CT of the abdomen and pelvis revealed no abnormalities. 

The patient was initially discharged with symptomatic treatment. One year later, he presented again with persistent flank pain of greater intensity, now associated with unquantified weight loss. On physical examination, his general condition was preserved. Deep palpation of the left flank revealed a poorly localized tenderness.

**Figure 1:**
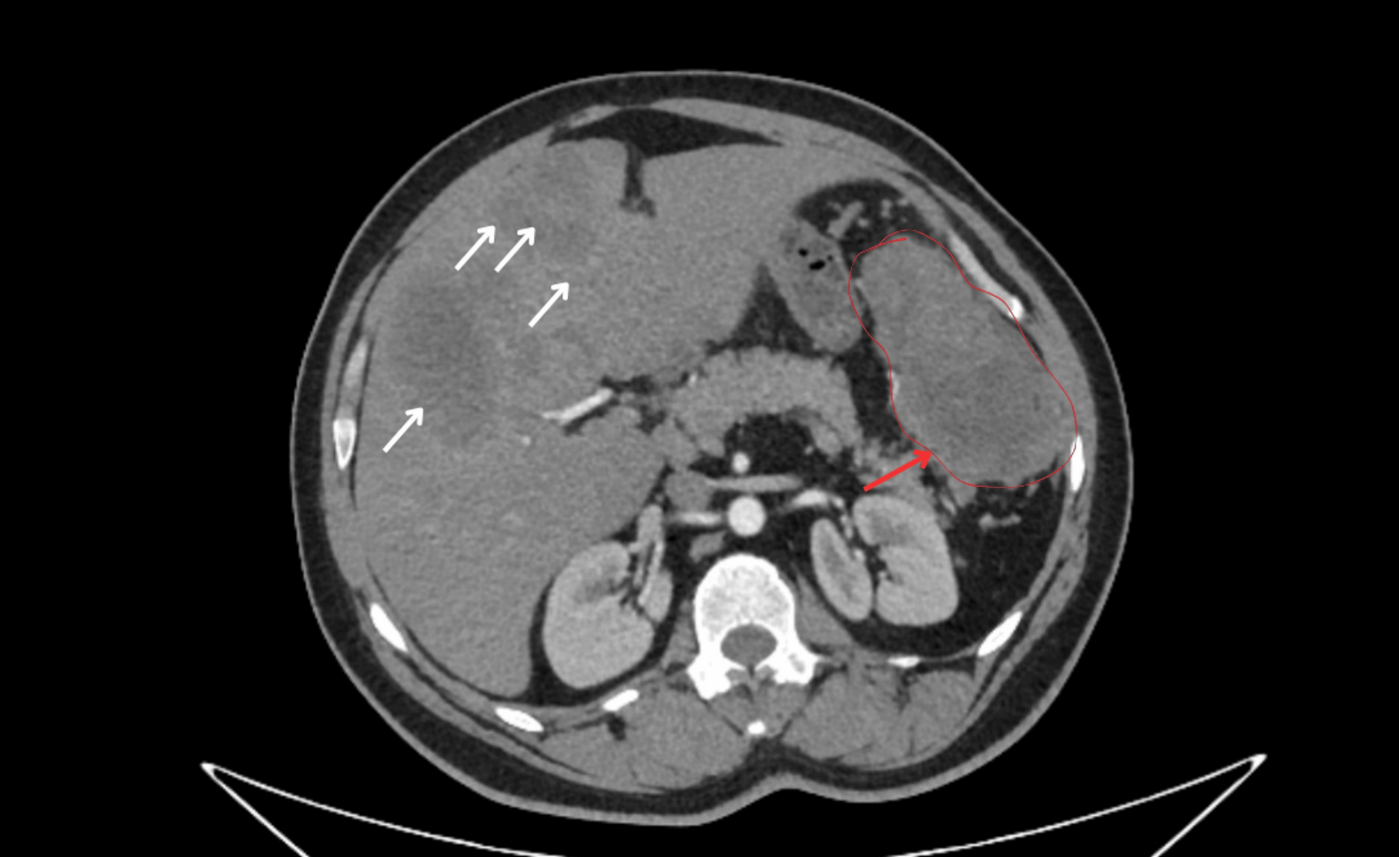
CT Imaging of the DSRCT peritoneal mass with liver metastases. DSRCT: desmoplastic small round cell tumor. Red arrow: Mass attached to the wall of the splenic flexure, measuring 113 × 61 mm. White arrows: Multiple peripheral, continuously enhancing lesions, consistent with liver metastases

**Figure 2:**
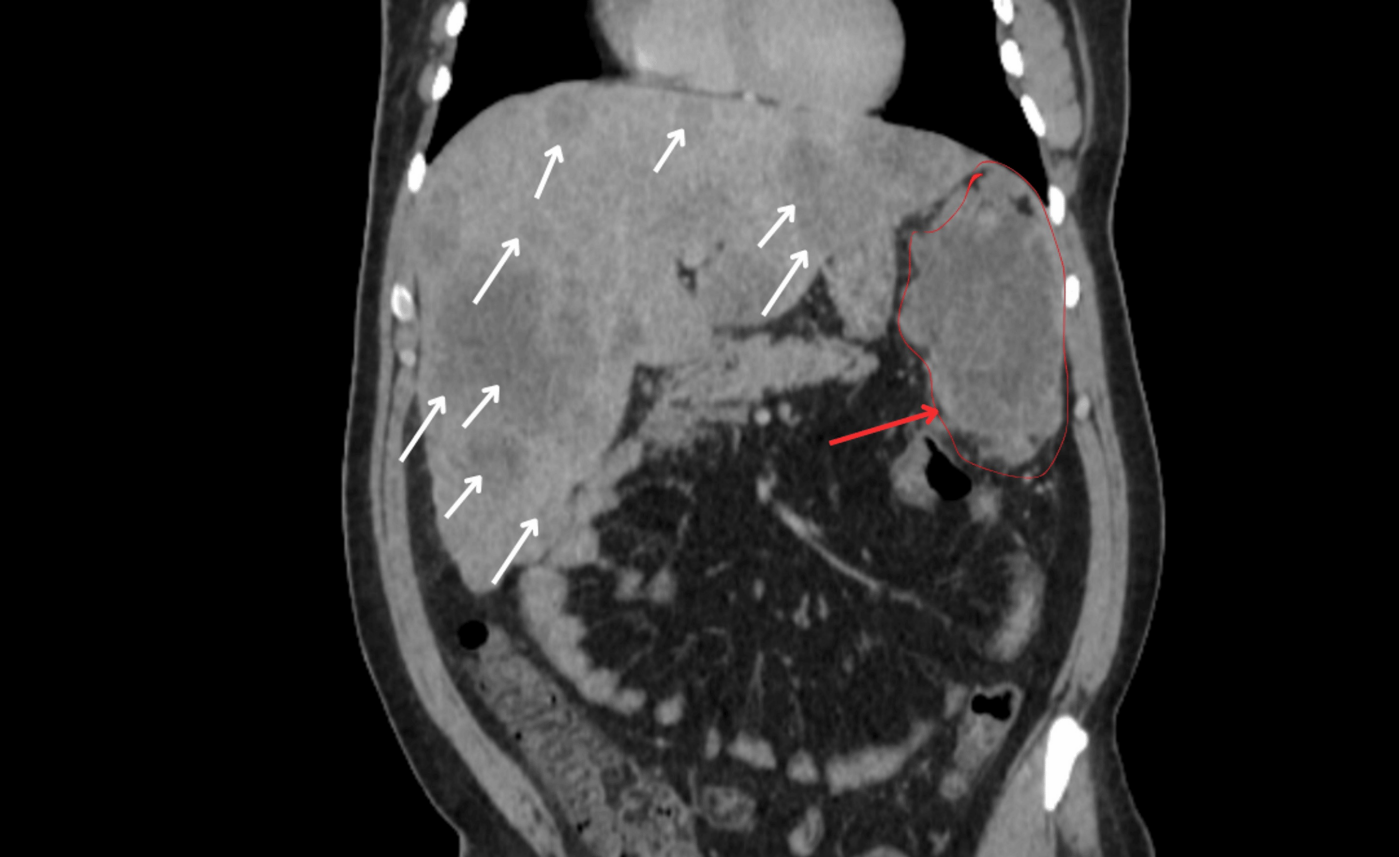
Coronal CT scan showing DSRCT peritoneal mass with hepatic involvement. DSRCT: desmoplastic small round cell tumor. Red arrow: Mass attached to the wall of the splenic flexure, measuring 113 × 61 mm. White arrows: Multiple peripheral, continuously enhancing lesions, consistent with liver metastases

An abdominal contrast-enhanced CT scan was indicated, which showed a left flank mass attached to the wall of the splenic flexure, measuring 113 × 61 mm, with a central necrotic component and heterogeneous enhancement after contrast injection. The lesion extended to involve the inferior pole of the spleen. Multiple anterior peritoneal nodules were also identified, the largest measuring 41 × 32 mm. The liver was of normal size but contained multiple peripheral, continuously enhancing lesions, consistent with liver metastases. No deep lymphadenopathy was observed (Figures 1, 2). 

A chest CT scan was performed and showed no abnormalities. An ultrasound-guided biopsy was performed. Histopathological analysis showed nests of small round cells embedded within a desmoplastic stroma. Immunohistochemistry demonstrated positivity for epithelial markers (cytokeratin, EMA), desmin (perinuclear dot pattern), WT1, and vimentin, while negative for CD34, CD117, and S100. The Ki-67 proliferation index was estimated at 70%. These findings confirmed the diagnosis of DSRCT. Molecular confirmation was performed by RT-PCR.

The patient's case was presented at the weekly multidisciplinary team meeting of the hospital for discussion. Given the extensive disease burden, particularly the hepatic and peritoneal involvement, a decision was made to refer the patient to the oncology team for palliative systemic therapy. Resection was not an option owing to the advanced extent of liver disease.

The palliative therapy plan was tailored to manage symptoms, control disease progression, and improve the patient's quality of life. A combination of chemotherapy and targeted therapy was considered, utilizing agents such as etoposide, ifosfamide, and vincristine, which have been part of standard treatment regimens for DSRCT, depending on individual patient response and tolerance. In addition to pain management and supportive care, with the aim of alleviating discomfort, managing side effects.

Later, the patient began experiencing abdominal pain that was resistant to analgesics at levels 1 and 2, eventually requiring morphine. The patient also started to develop significant cytolysis and cholestasis, which further complicated the continuation of the treatment.

The patient and their family were provided with clear information about the prognosis and the goal of therapy, and decisions were made collaboratively in alignment with the patient's wishes. The patient was placed on a palliative continued care plan, including analgesic and supportive treatment for four months. Chemotherapy was discontinued due to uncontrolled cytolysis. Patient consent was obtained for publication of the case details.

## Discussion

DSRCT is a rare and aggressive sarcoma that predominantly affects adolescent and young adult males, with a median age of 20-22 years [1,2,6]. Presentation in older patients is exceptional and emphasizes the need to consider DSRCT in the differential diagnosis of intra-abdominal masses even outside the usual age range [3]. The precise origin of DSRCTs in the gastrointestinal system remains unclear; however, they are believed to originate from primitive, multipotent cells capable of differentiating into multiple cell types. This is supported by the wide range of immunohistochemical markers expressed by DSRCT cells, which include epithelial, mesenchymal, and neural markers [7].

Clinically, DSRCT often presents with non symptoms or nonspecific symptoms such as abdominal pain, palpable mass, distension, or ascites, sometimes jaundice depending on the stage [4,5]. Symptoms, which depend on the tumor size and the location of the lesions, prompt further investigation through imaging studies.

In our patient, the initial CT and colonoscopy were normal, delaying diagnosis until the tumor became clinically apparent. This underlines the insidious nature of the disease and its potential to remain radiologically occult in early stages [6,8]. Early imaging may be normal or inconclusive. Tumors can initially present as small peritoneal nodules that are isodense relative to surrounding tissues, making them difficult to detect on standard CT scans [6,9]. Their growth is aggressive but often initially asymptomatic, and the desmoplastic stroma can mask lesions, particularly in pericolic or retroperitoneal locations. In some cases, these masses may even mimic other anatomical structures, such as an accessory spleen or adjacent organs, leading to potential misinterpretation [10]. Consequently, a normal initial scan does not rule out DSRCT, and persistent or worsening symptoms should prompt repeat imaging to identify the lesion once it has progressed [11,12].

Pathologically, DSRCT is characterized by nests of small round cells surrounded by a dense desmoplastic stroma [3,5]. Immunohistochemistry typically reveals polyphenotypic expression: epithelial (cytokeratin, EMA), mesenchymal (desmin, vimentin), and neural markers, along with nuclear WT1 positivity [9,10]. The EWSR1-WT1 gene fusion is the molecular hallmark, detectable by fluorescence in situ hybridization (FISH) or reverse transcription polymerase chain reaction (RT-PCR) [2,12]. Clinically and radiologically, DSRCT may mimic a variety of intra-abdominal masses, including sarcomas, lymphomas, desmoid tumors, and other small round cell neoplasms, particularly in the early stages. Because conventional imaging and morphology alone cannot reliably distinguish DSRCT from these entities, immunohistochemistry and molecular analysis are essential for definitive diagnosis.

The typical immunophenotypic profile of DSRCT is CK/EMA positive, desmin showing a perinuclear "dot-like" pattern, and nuclear WT1 (C-terminal) positivity. Definitive confirmation requires detection of the EWSR1-WT1 fusion via FISH, RT-PCR, or next-generation sequencing (NGS). This systematic approach allows accurate differentiation of DSRCT from other intra-abdominal neoplasms and guides appropriate management [7].

The following table summarizes the differential diagnoses of DSRCT in the GI tract, highlighting key features and the associated immunohistochemical markers for each condition (Table 2).

**Table 2:** Differential diagnosis of DSRCT of the GI tract. CD99: Cluster of Differentiation 99; EWS-FLI1 : fusion entre les gènes EWSR1 (Ewing Sarcoma Breakpoint Region 1) et FLI1 (Friend Leukemia Virus Integration 1; PAX8: Paired Box Gene 8; AFP: alpha-fetoprotein; β-hCG: beta-human chorionic gonadotropin; SALL4: Sal-like protein 4; PLAP: placental alkaline phosphatase; DOG1: discovered on GIST-1; GIST: gastrointestinal stromal tumor; LDH: lactate dehydrogenase; GI: gastrointestinal.

Tumor type	Key features	Markers
Ewing sarcoma	Aggressive bone tumor, primarily in children and young adults. Can present in the abdomen, with small round cells similar to DSRCT	CD99-positive, EWS-FLI1 fusion (EWSR1 + FLI1)
Alveolar rhabdomyosarcoma	Malignant soft tissue tumor originating from skeletal muscle cells, affecting children, with small round cells. Can involve the GI tract	Myogenin- and MyoD1-positive (muscle differentiation markers)
Neuroblastoma	Malignant tumor from neural crest cells, commonly found in infants. Often forms rosettes histologically and expresses neuroendocrine markers	Synaptophysin, chromogranin-positive
Small cell carcinoma	High-grade, aggressive cancer, often in the lungs but can arise in the GI tract. Composed of poorly differentiated small cells. Requires intensive chemotherapy	Chromogranin, LDH
Peritoneal mesothelioma	Malignant tumor of the peritoneum, typically associated with exposure to asbestos	Calretinin-positive
Lymphoma	Malignant neoplasm of lymphoid tissue that can involve the GI tract	CD45-positive
Serous carcinoma	A type of carcinoma, often affecting the ovaries but can also involve the GI tract	PAX8-positive
Germ cell tumors	Malignant tumors that can affect the GI tract. Often associated with testes or ovaries but can present in other locations	AFP, β-hCG, SALL4, PLAP-positive
GIST	A type of soft tissue tumor commonly arising in the GI tract, often with distinct molecular characteristics	CD117, DOG1-positive

DSRCT is an aggressive malignancy with poor prognosis, with overall five-year survival ranging from 15% to 30% despite intensive multimodal therapy [3,4]. Survival outcomes for DSRCTs depend on various factors, including the stage of the disease, treatment response, and recurrence. The timing of diagnosis and the presence of metastases at the time of diagnosis play a crucial role in determining the prognosis. Early detection of DSRCTs in the gastrointestinal tract is often challenging, as patients typically present with common GI symptoms that do not initially suggest malignancy. It is only when the tumor grows large enough to cause significant clinical signs and persistent symptoms that a more thorough medical evaluation and intervention are prompted.

Optimal management involves multimodal therapy, including intensive chemotherapy, cytoreductive surgery (CRS) when feasible, and radiotherapy [10].

Concerning chemotherapy, the mainstay of systemic therapy is intensive multi-agents often using Ewing sarcoma-type protocols such as VAC/IE (Vincristine, Doxorubicin, Cyclophosphamide alternating with Ifosfamide and Etoposide). Expected responses include partial or complete tumor shrinkage in most patients, facilitating surgery or symptom control, although long-term disease-free survival remains limited [9,12].

Hyperthermic intraperitoneal chemotherapy (HIPEC) is an emerging treatment approach, first introduced in 2004 and further explored in 2007, mainly for its effectiveness in eradicating microscopic disease following complete resection [13,14]. CRS with HIPEC may be considered in select patients [14]. Selection criteria include a low to moderate peritoneal disease burden (PCI); absence of extra-peritoneal metastases, particularly hepatic or pulmonary; and a patient who is fit for major surgery. The goal is maximal cytoreduction, evaluated using the Completeness of Cytoreduction (CC) score: CC-0, no visible residual tumor; CC-1, residual nodules <2.5 mm.

In our patient, CRS/HIPEC was not feasible due to diffuse hepatic metastases, precluding complete cytoreduction. Moreover, radiotherapy can also be used as consolidation after surgery or chemotherapy in localized disease, or for palliation in unresectable cases. Its role is limited in widespread disease but may help control pain or tumor bulk [15]. Given poor outcomes with conventional therapy, novel approaches are under investigation, focused on immune and biologically targeted therapies.

Potential strategies include targeting VEGF inhibition, androgen receptor activity, and agents that influence the connective tissue growth factor CCN2 (cellular communication network factor 2) and GD2 (glycosphingolipid receptors). Additionally, radioimmunotherapy (RIT) using the murine monoclonal antibody 131I-omburtamab, which targets the B7-H3 antigen, has advanced to phase 1 clinical trials [14,15]: anti-angiogenic agents (e.g., TKIs such as pazopanib, sunitinib), IGF-1R inhibitors, mTOR pathway modulators, and other targeted therapies and combination regimens under clinical trial like anlotinib, which has shown interesting results in recent trials [14,15].

Given advanced disease with diffuse hepatic involvement, our patient started receiving systemic palliative chemotherapy per Ewing sarcoma protocols. Surgical resection and HIPEC were not indicated, and radiotherapy was reserved for symptom control if needed and if the patient accepted. However, due to the hepatic complications and the alteration of general status, the treatment was put on hold.

A similar diagnostic challenge in adults has been reported in the literature [16]. This involved a 25-year-old man who presented with prolonged vague abdominal symptoms and extensive peritoneal carcinomatosis, underscoring the importance of considering DSRCT even in atypical adult presentations. Additionally, a case of DSRCT in a 51-year-old man with long-term survival following multimodal therapy illustrates that this tumor, although rare in older adults, can also occur outside the typical age range and may benefit from aggressive treatment, especially since it was still localized at diagnosis in this patient, allowing complete surgical excision [17].

## Conclusions

This case highlights several important points regarding DSRCT in adults. First, although DSRCT predominantly affects adolescents and young adults, our patient's age over 30 years underscores that it should not be excluded in older adults, and clinical suspicion must remain broad. Second, diagnostic vigilance is essential: persistent abdominal or flank symptoms warrant repeat and advanced imaging, as early-stage DSRCT may be radiologically occult.

Finally, accurate diagnosis relies on histopathology, including a complete immunohistochemical panel and confirmation of the EWSR1-WT1 fusion by molecular testing. In cases of peritoneal masses of uncertain etiology, early multimodal evaluation including imaging, immunohistochemistry, and molecular studies is key to timely diagnosis and appropriate management. In our patient, diffuse hepatic involvement precluded cytoreductive surgery and HIPEC, making systemic Ewing-type chemotherapy and analgesics the mainstay of therapy, with the hope of improving the patient's quality of life.
